# Infrared thermal imaging-based skin temperature response during cupping at two different negative pressures

**DOI:** 10.1038/s41598-022-19781-6

**Published:** 2022-09-15

**Authors:** Xulong Liu, Yanli Wang, Zhenying Wu

**Affiliations:** 1 Department of Biomedical Engineering, School of Computer and Communication Engineering, Northeastern University, Qinhuangdao, 066004 Hebei China; 2 Department of Acupuncture and Massage, Qinhuangdao Hospital of Traditional Chinese Medicine, Qinhuangdao, 066004 Hebei China

**Keywords:** Health care, Biomedical engineering, Imaging and sensing, Infrared spectroscopy

## Abstract

Cupping therapy can relieve muscle fatigue and pain after exercise by increasing blood flow at the treatment site, which may lead to dynamic changes of the local skin temperature. This study aimed to analyze the effect of cupping on local skin temperature under two different negative pressures using infrared thermography (IRT). Cupping therapy was performed on the forearms of 22 healthy subjects using the negative pressures of − 0.03 and − 0.04 MPa. IRT was used to record the dynamic changes in skin temperature before, during, and after cupping. Both cupping pressures induced a non-linear skin temperature response: temperature decreased first and then increased during cupping, while it first increased and then decreased after cupping. A significant difference was noted between the two negative pressure groups in the maximum temperature increment after cupping (*P* < 0.001). Compared with the basal temperature before cupping, the maximum increase in skin temperature after cupping in the − 0.03 and − 0.04 MPa groups was 0.92 and 1.42 °C, respectively. The findings of this study can lay the foundation evaluating the curative effect of cupping based on IRT and provide an objective reference for selecting the cupping negative pressure.

## Introduction

The ancient medical practice of cupping shows excellent effects in relieving the pain of limbs, head, neck, shoulders, and back, and has been used worldwide for thousands of years^1–3^. It uses cups to create negative pressure by burning oxygen or sucking out the air within the cup, so that the cup can be adsorbed on a specific part of the body surface to stimulate the body and achieve its purpose of relieving pain and treating and preventing diseases^4,5^. With the publication of studies concerning the application of cupping therapy on several well-known athletes, there has been a significant surge of interest in applying this therapy to relieve post-exercise fatigue and pain in the field of sports medicine^6^. The selection of cupping parameters has a great impact on the treatment effect, such as cupping pressure, duration and cup size. Recent studies show that cupping therapy changes local skin blood flow^7,8^. Furthermore, higher cupping pressure^9^, longer duration^9^ and larger cup size^10,11^ are more effective in increasing skin blood flow. These studies attempt to provide evidences for the cupping parameter selection. However, no specific guidelines have been formed yet^9^ and further research is needed.

Although there is no consensus about the underlying mechanisms of cupping therapy, studies show that pain relief with cupping therapy is associated with increased blood flow at the treatment site^9,12,13^. During cupping, the generated negative pressure results in the compression of the skin in contact with the cup body, resulting in swelling of the skin and the subcutaneous tissue within the cup, causing local skin microvascular relaxation and hence increased blood flow at the treatment site, and even partial capillary rupture to induce ecchymosis^14^. The reactive hyperemic pattern occurs after cupping therapy^11^, which could increase local blood flow^7,9,13^, reduce local lactate concentration^15,16^, accelerate tissue metabolism^16^, and improve soft tissue healing^9,12^. Therefore, cupping therapy may be helpful for relieving exercise fatigue and myofascial pain. Furthermore, studies demonstrated that different cupping pressures have different effects on skin blood flow responses^9^, which could offer a better understanding of the therapeutic mechanism of cupping therapy. For instance, Zhang et al. collected skin spectral data before and after cupping using a hyperspectral imaging camera, and found significant differences in the wavelength of the maximum change of skin spectrum after cupping under different negative pressures, indirectly indicating that the microcirculation blood perfusion of the skin increased significantly with the increase of negative pressure^17^. In addition, by comparing the color differences of the skin bruises after cupping under different negative pressures, Zhao et al. reported that with the increase of negative pressure there was an increase in skin microvascular blood flow, showing a darker color of the bruise after microvascular rupture^18^.

There are three factors which affect skin temperature: blood perfusion, tissue thermal conductivity and metabolic heat generation^19^. According to the skin heat transfer model, blood perfusion is the dominant factor affecting skin temperature^20^. When the ambient temperature and humidity are stable and the subjects have no physical activity, the change of skin temperature is mainly determined by skin blood flow dynamics^21^. The skin temperature could contain the information about the blood-flow signal existing in an earlier moment of time^21^, so it is possible to calculate skin blood flow indirectly through skin temperature measurements^21,22^. Previous studies showed that there was a significant positive correlation between skin temperature and skin blood perfusion in normal people (*r* = 0.742, *P* < 0.0001)^23^. Studies have reported that cupping therapy alters skin blood flow at the treatment site^7,8^, and cupping pressure is a key factor affecting skin blood flow^9^. Skin temperature is intimately associated with skin blood perfusion, and the changes in local blood flow may result from the interaction of skin microvascular contraction and relaxation^23^. Evaluating the time-course of skin temperature induced by different negative pressures is of great significance for the analysis of the dynamic response of skin blood flow to cupping. In general, there are two major approaches to measure human skin temperature, i.e., contact thermometry (thermocouple or thermistor) and radiation thermometry (infrared thermography). It should be noted that contact thermometry is inappropriate during cupping since the connecting wire of the temperature sensor may restrict the sorption of the cup body on the skin surface. On the other hand, infrared thermography (IRT) can record the temperature distribution of human skin by capturing the infrared radiation from the human body surface^24^. It has been extensively used in the detection and evaluation of vascular diseases^25^, pain^26^, breast cancer^27,28^, skin diseases^29^, sports injuries^30,31^, diabetic foot^32^, etc. In recent decades, IRT has been recognized as the most appropriate tool to assess the dynamic response of human skin temperature under external stimulation, due to its advantages of no contact, real-time measurement, and visualization of the temperature distribution^30^. Under external stimulation (cold stress^33–35^, electrostimulation^36^, acupuncture^37^, cupping^38,39^ etc.), there may be a time-dependent variation in the skin temperature of the various parts of the human body. The dynamic temperature response can be recorded in real time by IRT to facilitate further analysis of key parameters at each time point^40^, thus enabling a better understanding of homoiothermism and skin blood flow perfusion of the human body under various conditions. For example, Xu et al. conducted a study to observe the effect of cupping on skin temperature. They showed that local skin temperature was increased by 0.4 °C on average 10 min after cupping, suggesting that such increase may be correlated with the curative effect of cupping^38^. Cage et al. evaluated the effect of cupping on the skin temperature of the medial forearm, and observed significant differences in local skin temperature before, during, and after cupping, and in particular an obvious increase of skin temperature after cupping^39^.

These studies verified the feasibility of IRT applied to the mechanism of cupping, yet were characterized by two common limitations. First, there was no control of the negative pressure during cupping and no analysis of the influence of different negative pressures on skin temperature. Second, there was no analysis of the whole time-course of skin temperature response after cupping. Indeed, these studies only compared the skin temperature at several time points during the cupping process, without emphasizing the time-course of the whole skin temperature dynamic changes. To address these problems, negative pressure during cupping should be controlled during the analysis of the skin temperature regulatory response based on infrared imaging: this was precisely the main motivation of this study.

This study aimed to explore the dynamic changes of skin temperature during cupping under two different negative pressures. We hypothesized that a higher cupping pressure would be more effective on increasing skin temperature after cupping therapy compared with a lower pressure.

## Materials and methods

### Subjects

The subjects of this study were 22 healthy male graduate students from Northeastern University at Qinhuangdao in China, with mean age, body height, body weight, and body mass index of 24.30 ± 1.74 years, 174.10 ± 4.98 cm, 72.85 ± 8.86 kg, and 23.98 ± 2.22 kg/m^2^, respectively. All subjects had no smoking history, no skin damage on the medial forearms, and had not taken any drugs that might affect thermoregulation within 7 days before the trial. Meanwhile, subjects were required not to consume alcoholic or caffeinated drinks or apply any moisturizing or sunscreen products on their forearm skin within 12 h before the trial.

The protocol was approved by the Institutional Ethics Committee of Qinhuangdao Hospital of Traditional Chinese Medicine. This study complies with the Declaration of Helsinki and all methods were performed in accordance with relevant guidelines and regulations. All subjects signed informed consent after a full explanation of the protocol by our researchers.

### Equipment

**Figure 1:**
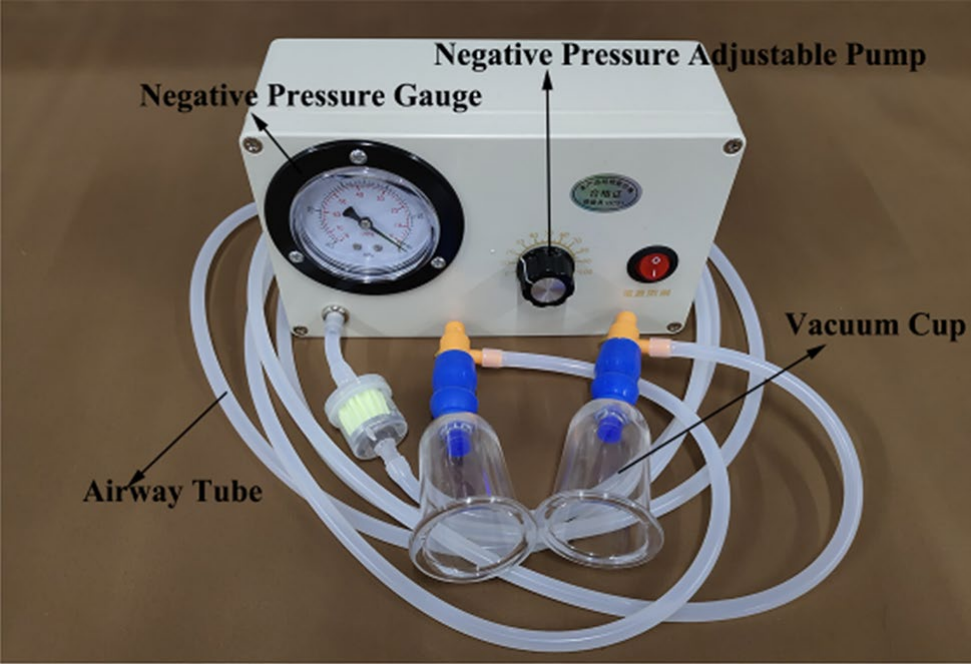
Structure of the electric vacuum-based cupping device.

An electric vacuum cupping device (MF-H96, Fujian Meifu Medical Instrument Co., Ltd., China) was used for cupping. The device consists of a negative pressure adjustable pump, a negative pressure gauge, and a vacuum cup, as shown in Fig. 1. The negative pressure produced by the air extracting pump ranged from − 0.01 to − 0.08 MPa. The vacuum cup is made of polycarbonate, with an inner diameter of 3.5 cm.

The thermal images of the subjects were collected by an IRSV-Net medical infrared thermal imager (type of inner camera core: FLIR Tau 336, FLIR Systems, Inc. Wilsonville, USA), whose detailed specifications are shown in Table 1. The emissivity of the thermogram was pre-set to 0.98^41,42^.

**Table 1:** FLIR Tau 336 main specifications.

Thermal camera core	Uncooled Microbolometer
Spectral band	7.5–13.5 µm
Scene temperature range	− 25 to 100 °C
FPA sensor size	336 × 256
Sensitivity (NEdT)	< 50 mK

### Experimental protocol

All subjects were scheduled to undergo the tests between 9 and 10 am to avoid the influence of the physiological rhythmic changes of skin temperature^43^. The experiment was completed in a controlled indoor environment with no significant air convection, with ambient temperature and humidity maintained at 25–26 °C and 45–55%, respectively.

**Figure 2:**
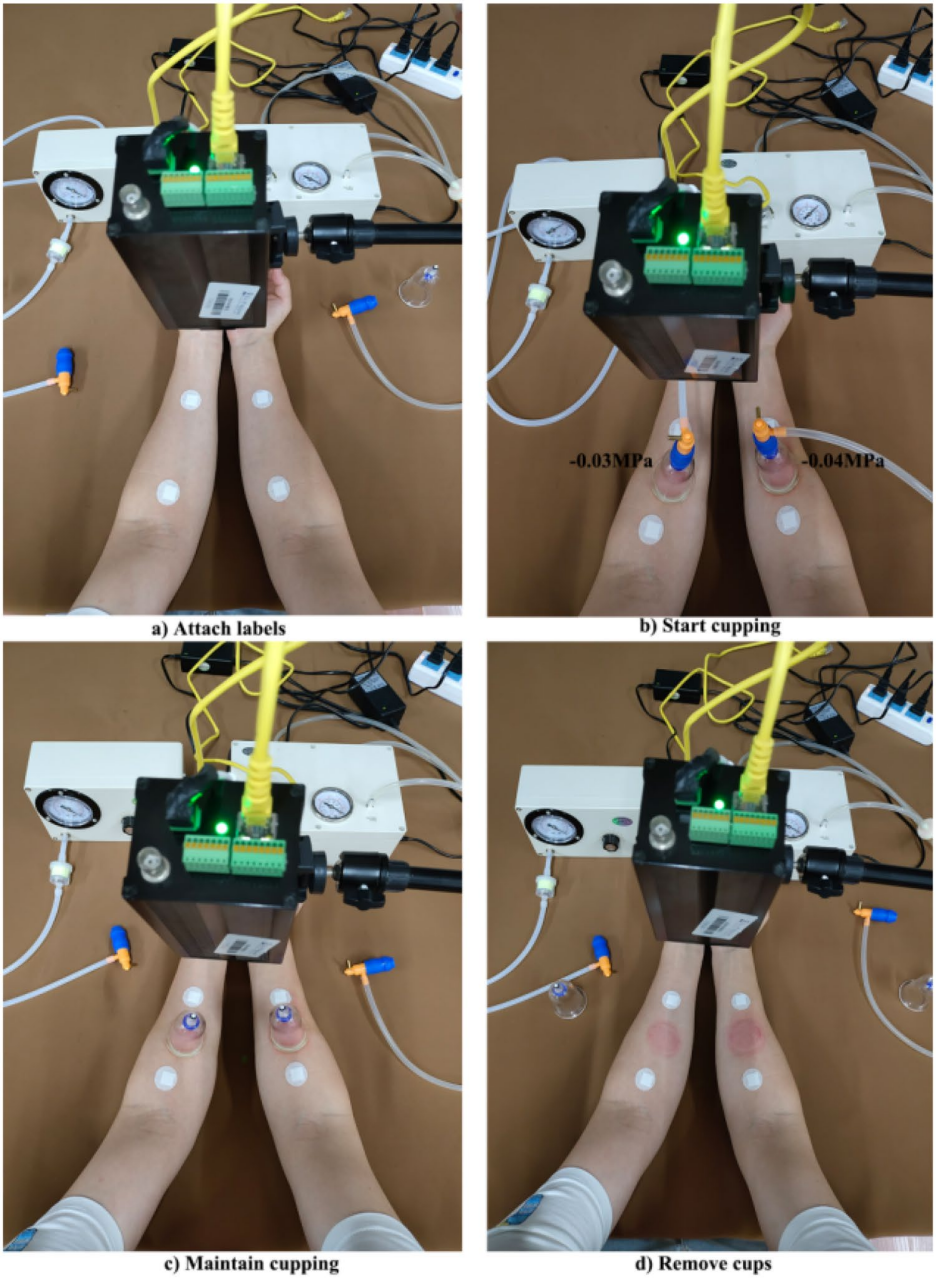
Complete process of the cupping test at two different negative pressures.

The cupping process and thermal image acquisition procedure consisted of the following steps:

1. Attach the labels (Fig. 2a). The cupping positions were marked on the medial side of the left and right forearms of the subjects, with one label attached to the upper and one to the lower area of the target region, as shown in Fig. 2a. After labeling, subjects were required to sit still for 15 min to adapt to the room temperature^43^.
2. Produce negative pressure (Fig. 2b). To start cupping, two electric vacuum cupping devices were simultaneously used to pump air, so that the negative pressure within the two cups was maintained at − 0.03 and − 0.04 MPa on the left and right forearm, respectively, for 20 s.
3. Maintain cupping (Fig. 2c). After pumping, the two vacuum cups were retained on the skin surface for three minutes based on the negative pressure within the cup.
4. Remove the cups (Fig. 2d). The subjects underwent continued observation for 30 min after cupping.

In this study, cupping pressures were randomly assigned to each subject's left and right forearms. Since the recruitment of subjects was random, the cupping pressure of each subject was assigned sequentially. In order of subject recruitment, the first subject used − 0.03 MPa on the left forearm and − 0.04 MPa on the right forearm, and the second subject used − 0.04 MPa on the left forearm and − 0.03 MPa on the right forearm. Then the subjects were assigned cupping pressures in this order.

The cupping duration was 3 min, and observation was continued for 30 min after cupping. Thermal images acquisition started 5 min before cupping and ended 30 min after cupping, with one image collected every 20 s. As shown in Fig. 2, in order to reduce viewing angle errors^34^, subjects were required to stretch their arms forward in a natural way with the camera of the thermal imager adjusted directly opposite to their arms.

The effects of the dominant and non-dominant sides on skin temperature response could be eliminated by cupping at the same site of the subjects. However, to the best of our knowledge, there are no clear guideline for the interval between cupping sessions. If the interval is too short, the legacy effect of the previous cupping could not be prevented. If the interval is too long, some factors affecting skin temperature^43^, such as environment, diet, exercise, metabolic rhythm, etc., are not easy to control. Therefore, cupping therapy was performed on the forearms of the subjects at the same time using the negative pressures of − 0.03 and − 0.04 MPa in this study, which could overcome the influences of the above factors. In order to eliminate the influence of the inherent temperature difference between the left and right forearms, two measures were adopted: (1) two different negative pressures were alternately assigned to the left and right forearms, and (2) the basal temperature of the forearms was subtracted when evaluating changes in skin temperature after cupping.

Negative pressure may be produced with two approaches, as described below: (1) by depleting the oxygen by igniting an alcohol cotton ball inside the cup; and (2) by pumping the air in the cup using an air pump. The latter method has been adopted by increasingly more studies^17,38,39,44,45^ since the former method may cause burns and cannot be operated by one person alone. A controllable negative pressure can be realized by sucking the air in the cup using the air pump, which can be adjusted to achieve − 0.02, − 0.03, − 0.04 and − 0.05 MPa during cupping. Cupping below − 0.02 MPa produces suction that is too small to effectively stimulate the human body, while cupping at − 0.05 MPa may induce pain and lead to intolerance in some subjects^18^. Accordingly, cupping at − 0.03 and − 0.04 MPa was performed for further comparative analysis in this study. Jan et al. suggested that a larger cup is more effective in increasing skin blood flow^11^ and improving muscle stiffness^10^. The selection of cupping size is related to the treatment site and should be as large as the anatomical area can accommodate^14^. Different from the study of Jan et al., the cupping location was the forearm in this study, which could accommodate a cupping cup with a maximum diameter of 3.5 cm. Because the entire medial forearm is a curved surface, a larger-caliber cupping cup (4.0 cm or 4.5 cm) cannot be tightly adsorbed on the skin during cupping, which affects the therapeutic effect.

Cupping therapy was usually performed on the forearm^39^, upper arm^9–11^ and back area^7,8^ in previous studies. In the use of infrared thermography (IRT), the lens is required to be perpendicular to the temperature measurement area to reduce the angle error^44^. In this study, the skin temperature was measured from 5 min before cupping to 30 min after cupping. The whole process would last for a long time. Accordingly, it was difficult to select an appropriate posture for the subjects to maintain the fixed angle between the cupping position and the thermal camera lens if the cupping location was chosen on the upper arm. For cupping on the back, subjects can use prone posture to make the IRT perpendicular to the cupping location. But long time prone will make the chest compression, which make some subjects feel uncomfortable and make it difficult to maintain a fixed position. Meanwhile, the subjects need to expose their upper body for a long time when cupping is applied on the back. Skin temperature may have additional changes due to the influence of chest compression and environment, which might potentially affect the skin temperature response after cupping. Therefore, in this study, the forearm was selected as the cupping location. The advantage is that the subjects can choose sitting posture, and it is easy to adjust and maintain the thermal image lens perpendicular to the cupping location during long-term temperature measurement.

### Thermographic analysis

**Figure 3:**
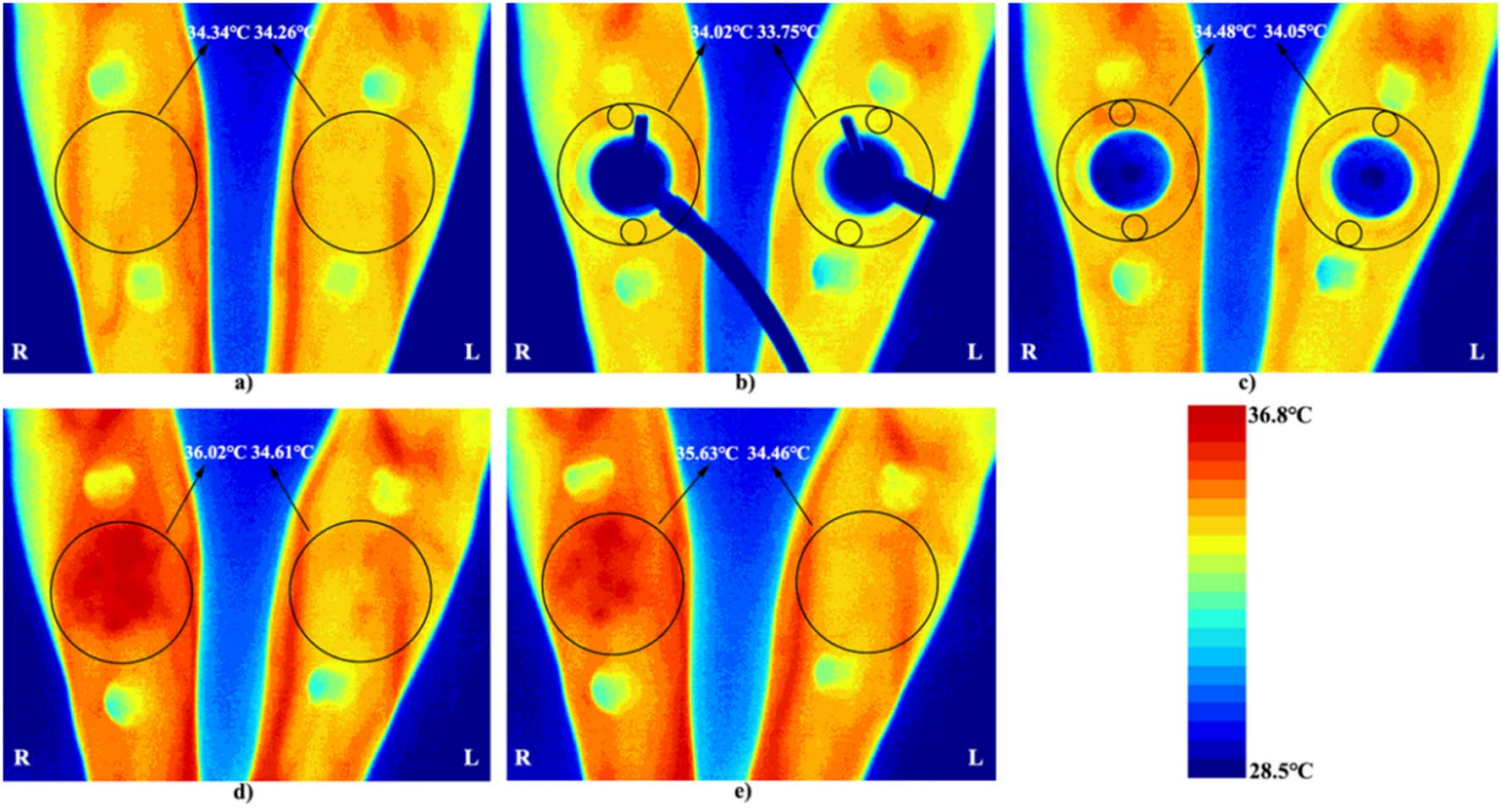
Measurement of the skin temperature of the cupping site at different stages of the experiment using IRT. ROIs are marked with black circles. (**a**) The basal temperature before cupping is measured. (**b**) Cupping is started at − 0.03 MPa on the left and − 0.04 MPa on the right. The mean temperature in the ROI is represented by the average temperature of two small circular regions. (**c**) Cupping is maintained. (**d**) 500 s after cupping. (**e**) 1800 s after cupping. *R* right, *L* left.

To analyze the effect of cupping under different negative pressures on local skin temperature, the temperature changes of the region of interest (ROI) before and after cupping were recorded. Since the skin adjacent to the cupping is also pulled toward the center of the cupping, the region of interest (ROI) for temperature measurement in Fig. 3 is larger than the projected area of the cupping cup, including the skin around the cupping. First, five minutes before cupping, the average temperature of the ROI was measured to determine the basal temperature, as shown in Fig. 3a. Second, after the start of the cupping, since the cup body prevented measuring the temperature directly under the cup, the average temperature of two small local areas in the upper and lower parts around the cup within the ROI was used to represent the average temperature of the whole ROI (Fig. 3b,c). Finally, after the end of cupping, the average temperature of the ROI was measured continuously for 30 min (Fig. 3d,e) to observe the recovery of skin temperature after stimulation under different negative pressures.

The reliability of temperature measurements by IRT in humans has been shown to vary with the location of the ROI^46^. In view of this, the method of labeling around the ROI can significantly improve intra and inter-rater reliability^47^. Consequently, as shown in Fig. 2, two labels were attached around the ROI of the subjects before cupping. The labels are made of thermal insulation materials. Hence, there was a significant difference between the surface temperature of these labels and that of human skin, which could be easily seen in infrared thermogram (see e.g. Fig. 3), thus improving the reliability of IRT analysis. Furthermore, in multiple studies of sports medicine, the maximum temperature is commonly used to represent the temperature distribution of a ROI^48–50^. It is suitable in case of uneven distribution of hot or cold spots in the infrared thermogram. However, in our study, such phenomenon was not observed in the ROI temperature distribution shown by the infrared thermogram after cupping (Fig. 3). Therefore, only the average temperature value was selected to represent the temperature characteristics of the ROI.

**Figure 4:**
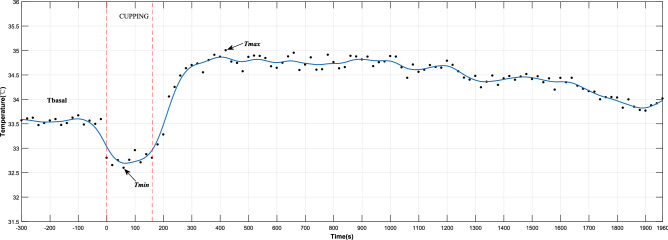
Time-dependent skin temperature changes induced by negative pressure (− 0.04 MPa) in a representative subject. The black dots represent the temperature series sampled in the experiment. The blue curve is obtained from these data using smooth spline fitting, and is divided into three stages by the red dash-dotted lines: basal stage, cupping stage and recovery stage. In these three stages, the three parameters (T_basal_, T_min_, and T_max_) indicated by the arrows were used to quantify the dynamic change limits of skin temperature. Table 2 shows the detailed definition of these parameters.

The dynamic change of skin temperature induced by negative pressure (Fig. 4) was subdivided into three stages. The first stage corresponded to the five minutes before cupping. The second stage lasted for three minutes, during which cupping was started under the negative pressures of − 0.03 and − 0.04 MPa on the medial side of the left and right forearms, respectively, of the subjects. The third stage was the recovery period after cupping, during which subjects were observed for 30 min. In order to quantitatively describe the time-course of skin temperature, based on the typical appearance of the temperature time-course (Fig. 4), six representative parameters were defined, their abbreviations, definitions, and calculations being shown in Table 2 and Fig. 4.

**Table 2:** Representative parameters describing the skin temperature time course.

Abbreviation	Definition	Units
Basal temperature (T_basal_)	The mean value of the temperature series sampled before the test	°C
Minimum temperature (T_min_)	The minimum value of the temperature series sampled during cupping	°C
Maximum temperature (T_max_)	The maximum value of the temperature series sampled after cupping	°C
Time to T_max_	Time to reach the maximum temperature	s
Maximum temperature increment (∆T_max_)	The maximum value of the difference between the temperature series and the basal temperature (T_max_ − T_basal_)	°C
Maximum temperature decrement (∆T_dnc_)	The difference between the basal temperature and the minimum value of the temperature series sampled during cupping (T_basal_ − T_min_)	°C

T_max_ indirectly reflects the maximum skin blood flow at the treatment site after cupping. Time to T_max_ indicates the time to peak skin blood flow after cupping. T_min_ indirectly reflects the maximum blood flow stagnation caused by negative pressures during the cupping process. Based on T_max_ and T_min_, maximum temperature increment (∆T_max_) and maximum temperature decrement (∆T_dnc_) eliminate the impact of individual basal temperature, which more effectively reflect the skin temperature response of cupping therapy and indirectly describe the changes in skin blood flow.

### Statistical analysis

Data were expressed as mean ± standard deviation (SD), and the Shapiro–Wilk's test was used to verify the normality of their distribution. A one-way ANOVA with repeated measures was applied to assess the intra-group skin temperature changes before, during, and after cupping. Tukey's multiple comparisons test was used as the post hoc test. In addition, the independent samples *t*-test was used to compare the difference in the skin temperature parameters between the two negative pressure groups (− 0.03 and − 0.04 MPa). *P*-values < 0.05 were considered statistically significant. Statistical analysis was performed using SPSS 22.0 statistical software (IBM, Armonk, NY, USA).

## Results

**Figure 5:**
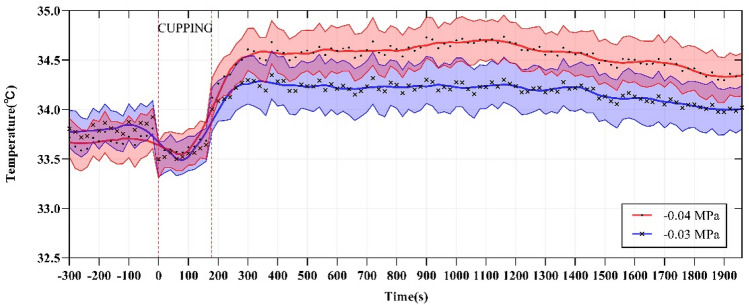
The mean temperature series of all temperature measurements collected during the experiment, averaged for each time point. The curve was obtained from these average temperature values (mean ± SEM) based on smooth spline fitting.

**Figure 6:**
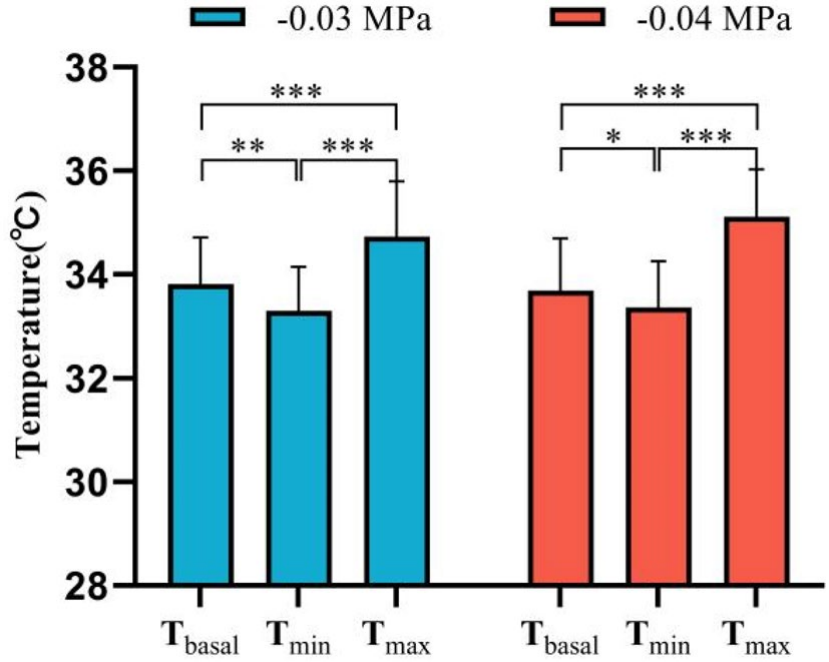
Comparison of T_basal_, T_min_, and T_max_ (mean ± SD). *P < 0.05; **P < 0.01; ***P < 0.001.

Figure 3 shows the typical thermal images of each stage during the test. As shown in this figure, under the two different negative pressures, the skin temperature of the medial region in the left and right forearms of the subjects first decreased, then increased, and finally decreased again. For further quantitative analysis, two curves were fitted with all the temperatures collected during the whole experiment, as shown in Fig. 5. The two curves were divided into the three stages, i.e., pre-experiment (− 300 to 0 s), during cupping (0–180 s), and after cupping (180–1960 s). Before the experiment, skin temperature showed minor fluctuations, and the average of all temperatures at this stage was defined as the basal temperature (T_basal_) and used in the analysis of the subsequent dynamic changes of skin temperature. During cupping, the skin temperature first decreased and then began to rise after reaching its minimum (T_min_). After cupping, it continued to rise, reached a peak (T_max_), and then decreased gradually after some time. A comparison of key parameters (T_basal_, T_min_ and T_max_) at the three stages is shown in Fig. 6. One-way repeated measures ANOVA revealed significant differences among the three parameters (− 0.03 MPa, *P* < 0.001; − 0.04 MPa, *P* < 0.001).

**Figure 7:**
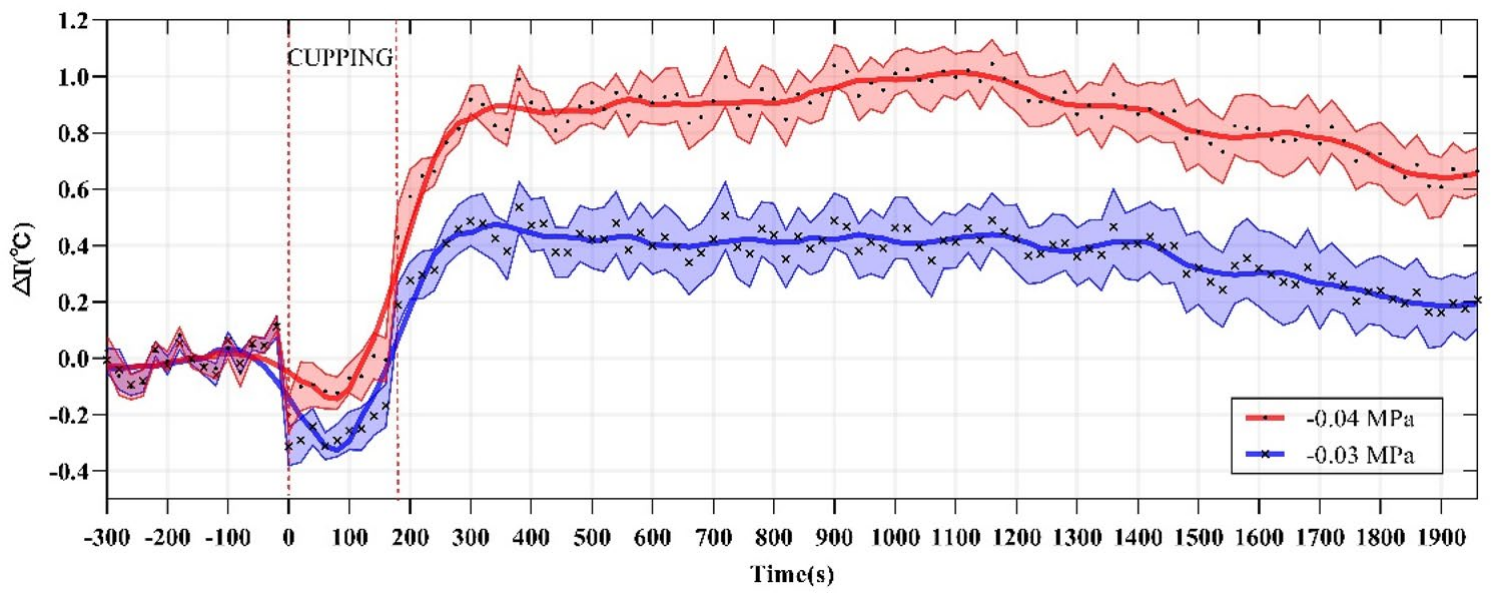
The average temperature variation of each time point with respect to the basal temperature during the test. The dots represent the average temperature difference, and the curves are obtained from these average temperature values using smooth spline fitting.

**Figure 8:**
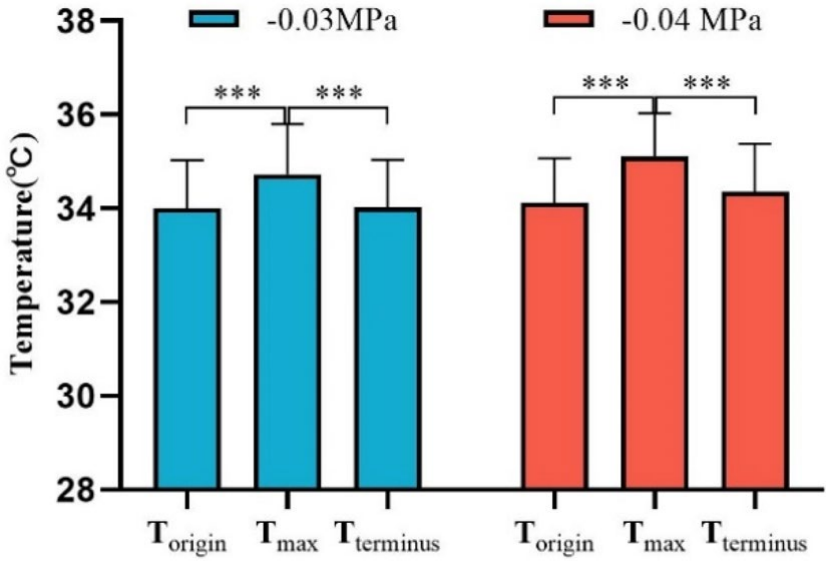
Comparison of the temperature values (original point, peak value and terminal point) at three representative moments after cupping (mean ± SD). ***P < 0.001.

**Figure 9:**
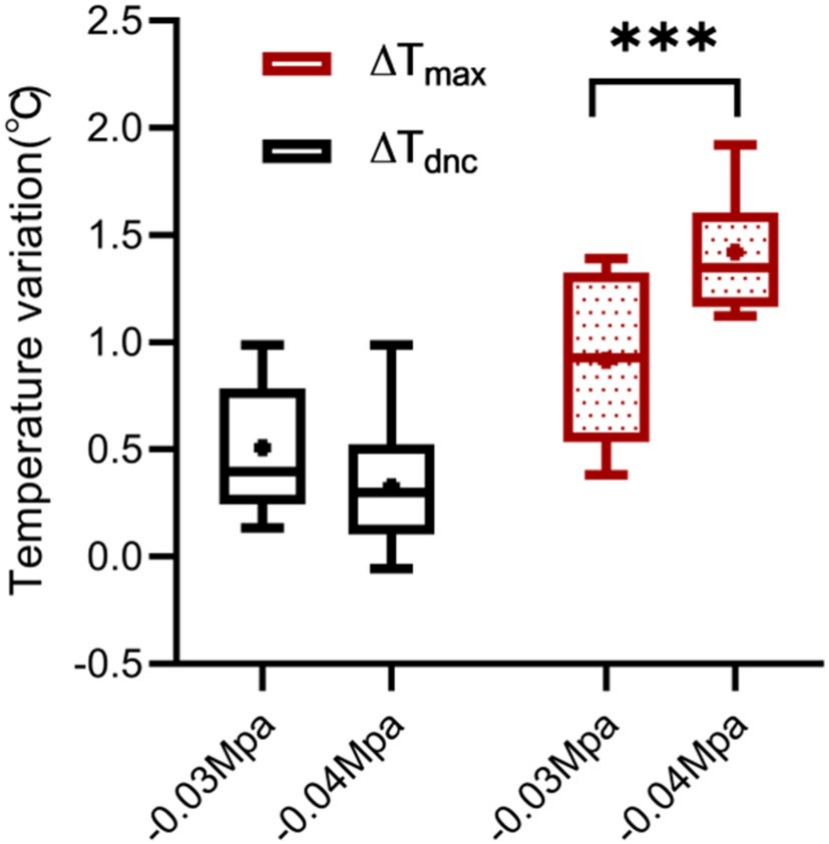
Comparison between ∆T_dnc_ and ∆T_max_ in the two negative pressure groups. The dot represents the mean, and the horizontal lines in the boxplot represent, from bottom to top, the minimum, first quartile, median, third quartile, and maximum. ***P < 0.001.

In order to compare the effect of cupping on local skin temperature during cupping under − 0.03 and − 0.04 MPa, the six representative parameters were calculated, the results being shown in Table 3. Specifically, after cupping, the maximum (T_max_) of the skin temperature induced by two different negative pressures (Fig. 6) were 34.73 and 35.11 °C on average, respectively. In addition, to eliminate the impact of the difference in basal temperature between the left and right forearms on subsequent skin temperature response, T_basal_ was subtracted from the temperature series collected during the experiment to obtain the time-course curve of the temperature change (Fig. 7). The temperature values (original point, peak value and terminal point) at three representative moments after cupping were compared in Fig. 8, and the Maximum temperature increment values (∆T_max_) of skin temperature after cupping at − 0.03 and − 0.04 MPa were compared in Fig. 9. After cupping, the ∆T_max_ of the ROI was 0.92 and 1.42 °C on average compared with that before cupping, respectively, and the difference was statistically significant (Fig. 9).

**Table 3:** Comparison of the parameter values extracted from skin temperature time-courses (mean ± SD) between the two groups.

Parameter	− 0.03 MPa	− 0.04 MPa	*P* value
T_basal_, °C	33.81 ± 0.90	33.69 ± 1.01	0.681
T_min_, °C	33.30 ± 0.85	33.36 ± 0.89	0.818
T_max_, °C	34.73 ± 1.07	35.11 ± 0.92	0.213
Time to T_max_, s	598 ± 350.55	580 ± 263.82	0.850
∆T_max_, °C	0.92 ± 0.42	1.42 ± 0.29	0.0005
∆T_dnc_, °C	0.51 ± 0.3	0.33 ± 0.31	0.059

## Discussion

This study provides the thermographic evidences of the effect of different cupping pressure on skin temperature responses. The results showed a non-linear skin temperature response throughout the cupping process. Specifically, skin temperature around the treatment site first decreased and then increased during cupping, and then showed the opposite behavior, namely an increase followed by a decrease and gradually returned to stability, after cupping. Moreover, a higher cupping pressure (− 0.04 MPa) is more effective on increasing skin temperature compared to a lower pressure (− 0.03 MPa).

Cupping therapy has been used for thousands of years to relieve muscle fatigue and pain. However, the mechanism of the cupping effect is currently under active investigation. Existing studies support the increase of blood flow at the cupping site as a reasonable explanation^12,13^. Various methods for the direct or indirect measurement of blood flow have been applied to observe its changes after cupping, including laser Doppler blood flow imaging^9,44^, near-infrared spectroscopy^7,45^, hyperspectral imaging^17^, IRT^38,39^, etc. Previous studies have shown that cupping pressure is an important factor affecting skin blood flow response^9,12^. The skin temperature represents another characteristic of skin blood flow^21^. Therefore, this study was conducted to analyze the effect of cupping on skin temperature under different negative pressures using IRT.

As shown in Fig. 5, an analysis was conducted focusing on the time-dependent change of skin temperature in the whole ROI. The results showed a nonlinear trend of changes in skin temperature caused by cupping. Specifically, skin temperature first decreased and then increased during cupping, and then showed the opposite behavior, namely an increase followed by a decrease, after cupping. The reasons behind this may be that, at the beginning of cupping, a rapid increase in the negative pressure tightened the skin by the edge of the cup, resulting in a decrease of local blood flow leading to temporarily decreased skin temperature. After the stabilization of the negative pressure, the pressure difference between the skin and the subcutaneous capillary in the cup may expand the space between the subcutaneous tissues, which would lead to the relaxation and even partial rupture of skin capillaries to promote the increase of local blood flow, as indicated by the rapid rise of skin temperature^14^. However, relevant studies only reported that the local skin temperature increased after cupping^38,39^. The reason for the inconsistency between these conclusions is the different observation time after cupping. The observation time after cupping adopted by Xu et al.^38^ and Cage et al.^39^ were 5 min and 10 min respectively. In this study, the time to the peak temperature after cupping at − 0.03 MPa was 598 s on average. Therefore, the observation time after cupping set in the above studies^38,39^ could be too short to observe the decreasing phase of skin temperature after cupping.

In this study, the dynamic change of skin temperature during cupping under two different negative pressures (Fig. 5) was divided into three stages, namely before, during, and after cupping. In each stage, one representative parameter was selected for comparative analysis to quantify the time-course of skin temperature changes. Before cupping, there was a slight fluctuation in the skin temperature, and all temperatures at this stage were averaged as the basal temperature (T_basal_) to compare and analyze dynamic changes of skin temperature induced by cupping under different negative pressures. During cupping, the skin temperature first decreased and then increased, and the minimum (T_min_) was selected as the main feature. After cupping, the skin temperature showed a trend of initial increase and later decrease, and the maximum value (T_max_) was taken as the representative parameter for this stage. Figure 6 shows the comparison among these key parameters. Statistically significant differences were found among the key parameters of the three stages in the process of cupping, under both − 0.03 and − 0.04 MPa. Further pair-wise comparisons indicated significant differences between T_basal_ and T_max_, further confirming that skin temperature rises after cupping. The results suggest that the two different negative pressures used in our experiment can produce effective stimulation on the human body to increase blood flow at the treatment site.

On the whole, the skin temperature after cupping first increased and then gradually decreased to become stable. There were significant differences in the temperature values at three representative moments after cupping (*P* < 0.001), while there were no significant differences in the temperature values at the original and terminal points (Fig. 8). This result indirectly indicates that the change of skin blood flow in the treatment site after cupping is a process of significant increase at first and gradual recovery later.

As shown in Table 3, the effects on skin temperature of cupping under − 0.03 and − 0.04 MPa were clearly different. Before cupping, no significant difference was observed in the T_basal_ of the ROI between groups, while a significant difference was detected in ∆T_max_ after cupping. This result supports a more robust rise of skin temperature using cupping under − 0.04 MPa than that under − 0.03 MPa. This is due to the fact that cupping under − 0.04 MPa can produce stronger suction than under − 0.03 MPa, resulting in an extensive relaxation or rupture of subcutaneous capillaries, and hence greater local blood flow, demonstrated by a more significant skin temperature rise. Additionally, there was no significant difference in the time to T_max_ between groups, which may be attributed to differences in the subjects’ tolerance of negative pressures. After cupping under − 0.03 and − 0.04 MPa, the time values to peak by two different negative pressures were 598 and 580 s on average, respectively, but there was no significant difference. It may be caused by the differences of subjects' subcutaneous vascular and fat distribution and tolerance of negative pressures.

The average temperature difference between the left and right forearms of healthy subjects has been reported to be 0.12 °C^51^. To reduce the impact of this difference on the experimental results, the value of T_basal_ was subtracted from the temperature series collected during the process. Finally, the time-course of the skin temperature variation was obtained, as shown in Fig. 7. The variations in skin temperature before cupping was relatively small, basically within 0.2 °C. However, as shown in Fig. 9, the average values of ∆T_max_ were 0.92 and 1.42 °C after cupping under − 0.03 and − 0.04 MPa, respectively. These results confirm that the use of cupping at − 0.04 MPa can induce a stronger stimulation of the human skin than cupping at − 0.03 MPa, resulting in a more significant rise of skin temperature.

The skin temperature first increased to a peak and then gradually decreased after cupping. There were no significant differences between the two negative pressure groups in the duration of the decreasing phase or in the temperature values at the last moment. This is mainly because the cupping pressure and duration used in the study would not cause skin damage after cupping^18^, and the temperature decreasing phase after cupping is mainly a self-recovery process of human skin, which is less affected by cupping pressure.

This study was conducted to analyze the effect of different cupping pressure on skin temperature responses using infrared thermography (IRT). A significant difference was noted in skin temperature before and after cupping (*P* < 0.001), and the results showed a greater effect on the increase of skin temperature under − 0.04 MPa than that under − 0.03 MPa (*P* < 0.001). Previous studies show that cupping therapy can improve regional skin blood flow^7,9^, and the change of skin temperature can indirectly reflect the change of skin blood flow^21,39^. Therefore, this research could verify the cupping effect on skin blood flow from a new perspective. In addition, Jan et al. studied the effect of various cup size of cupping therapy on skin blood flow response and found that the cups with larger diameters were more effective in increasing skin blood flow^10,11^. Therefore, the effect of the cupping size of cupping therapy on skin temperature response needs to be further studied.

Our study has several limitations. First, the most obvious limitation is that skin temperature cannot fully reflect the skin blood flow. In future studies, it is considered to measure skin blood flow after cupping using laser Doppler flowmetry or near-infrared spectroscopic (NIRS) sensor, and make correlation analysis about skin temperature and blood flow, so as to study the therapeutic mechanism of different cupping pressures from multiple perspectives. Second, due to the shielding of the cupping cup, the study cannot directly measure skin temperature under the cup by IRT. Rather than the temperature of the entire ROI, the study could only use the temperature of the adjacent area around the cup for analysis during cupping (Fig. 3b). This problem may be addressed by restructuring the cup body in future studies. Last, the study focused on the effect of different cupping pressures on skin temperature response, and included only males. Therefore, the effects of cupping duration, cup size, and other factors on skin temperature change need to be further analyzed. Moreover, female subjects need to be supplemented in future studies to ensure that statistical power has been achieved.

## Conclusions

In conclusion, cupping under two different negative pressures of − 0.03 and − 0.04 MPa can significantly increase local skin temperature at the treatment site. The nonlinear time profiles of skin temperature response were observed during the experiment in the cupping process under the two values of negative pressure. Local skin temperature revealed a trend of decrease followed by increase during cupping, and increase followed by decrease after cupping; moreover, cupping under − 0.04 MPa had a more significant effect on local skin temperature than that under − 0.03 MPa. Taken together, these results provide detailed thermographic data of the skin temperature response to the cupping process under different negative pressures, indirectly verify the local increase of blood flow after cupping therapy, and provide useful reference data for negative pressure selection during cupping.

## Data Availability

The datasets used and analyzed during the current study are available from the corresponding author on reasonable request.

## References

[1] Rozenfeld E, Kalichman L (2016). New is the well-forgotten old: The use of dry cupping in musculoskeletal medicine. J. Bodyw. Mov. Ther..

[2] Mehta P, Dhapte V (2015). Cupping therapy: A prudent remedy for a plethora of medical ailments. J. Tradit. Complement. Med..

[3] Seo J, Chu H, Kim C-H, Sung K-K, Lee S (2021). Cupping therapy for migraine: A PRISMA-compliant systematic review and meta-analysis of randomized controlled trials. Evid. Based Complement. Altern. Med..

[4] Arce-Esquivel AA, Warner BJ, Gallegos DM, Cage SA (2017). Effect of dry cupping on vascular function among young individuals. Int. J. Health Sci..

[5] Cao H (2014). Cupping therapy for acute and chronic pain management: A systematic review of randomized clinical trials. J. Tradit. Chin. Med. Sci..

[6] Bridgett R, Klose P, Duffield R, Mydock S, Lauche R (2018). Effects of cupping therapy in amateur and professional athletes: Systematic review of randomized controlled trials. J. Altern. Complement. Med..

[7] Kim S, Kim E, Jung G, Lee S, Kim JG (2019). The hemodynamic changes during cupping therapy monitored by using an optical sensor embedded cup. J. Biophotonics.

[8] Gao C, Wang M, He L, He Y, Li T (2019). Alternations of hemodynamic parameters during Chinese cupping therapy assessed by an embedded near-infrared spectroscopy monitor. Biomed. Opt. Express.

[9] Wang X (2020). Effect of pressures and durations of cupping therapy on skin blood flow responses. Front. Bioeng. Biotechnol..

[10] Jan Y-K (2021). Using elastographic ultrasound to assess the effect of cupping size of cupping therapy on stiffness of triceps muscle. Am. J. Phys. Med. Rehabil..

[11] He X (2021). Using reactive hyperemia to investigate the effect of cupping sizes of cupping therapy on skin blood flow responses. J. Back Musculoskelet. Rehabil..

[12] Lowe DT (2017). Cupping therapy: An analysis of the effects of suction on skin and the possible influence on human health. Complement. Ther. Clin. Pract..

[13] Wei LIU, Piao S, Meng X, Wei L (2013). Effects of cupping on blood flow under skin of back in healthy human. World J. Acupunct. Moxib..

[14] Tham LM, Lee HP, Lu C (2006). Cupping: From a biomechanical perspective. J. Biomech..

[15] Akimov EB, Son’kin VD (2011). Skin temperature and lactate threshold during muscle work in athletes. Hum. Physiol..

[16] Al-Bedah AMN (2019). The medical perspective of cupping therapy: Effects and mechanisms of action. J. Tradit. Complement. Med..

[17] Tian Y (2016). Color spectrum of cupping mark detected by hyperspectral camera: A preliminary observation. Zhongguo Zhen jiu Chin. Acupunct. Moxib..

[18] Zhao XX, Tong BY, Wang XX, Sun GL (2009). Effect of time and pressure factors on the cupping mark color. Zhongguo Zhen jiu Chin. Acupunct. Moxib..

[19] Swain ID, Grant LJ (1989). Methods of measuring skin blood flow. Phys. Med. Biol..

[20] Wu S, Lin W, Xie S (2008). Skin heat transfer model of facial thermograms and its application in face recognition. Pattern Recogn..

[21] Sagaidachnyi AA, Fomin AV, Usanov DA, Skripal AV (2017). Thermography-based blood flow imaging in human skin of the hands and feet: A spectral filtering approach. Physiol. Meas..

[22] Bornmyr S, Svensson H (1991). Thermography and laser-Doppler flowmetry for monitoring changes in finger skin blood flow upon cigarette smoking. Clin. Physiol..

[23] Schlager O (2010). Correlation of infrared thermography and skin perfusion in Raynaud patients and in healthy controls. Microvasc. Res..

[24] Ring FJ (2014). Pioneering progress in infrared imaging in medicine. Quant. Infrared Thermogr. J..

[25] Mi B, Song J, Hong W, Zhang W, Wang Y (2019). Evaluation method of infrared thermography on children with idiopathic thrombocytopenic purpura: Preliminary. Infrared Phys. Technol..

[26] Herry CL, Frize M (2004). Quantitative assessment of pain-related thermal dysfunction through clinical digital infrared thermal imaging. Biomed. Eng. Online.

[27] Gogoi UR, Majumdar G, Bhowmik MK, Ghosh AK (2019). Evaluating the efficiency of infrared breast thermography for early breast cancer risk prediction in asymptomatic population. Infrared Phys. Technol..

[28] Lubkowska A, Chudecka M (2021). Thermal characteristics of breast surface temperature in healthy women. Int. J. Environ. Res. Public Health.

[29] Magalhaes C (2019). Distinguishing melanocytic nevi from melanomas using static and dynamic infrared thermal imaging. J. Eur. Acad. Dermatol. Venereol..

[30] Gomez-Carmona P, Fernandez-Cuevas I, Sillero-Quintana M, Arnaiz-Lastras J, Navandar A (2020). Infrared thermography protocol on reducing the incidence of soccer injuries. J. Sport Rehabil..

[31] Chudecka M, Lubkowska A, Leznicka K, Krupecki K (2015). The use of thermal imaging in the evaluation of the symmetry of muscle activity in various types of exercises (symmetrical and asymmetrical). J. Hum. Kinet..

[32] Adam M (2017). Computer aided diagnosis of diabetic foot using infrared thermography: A review. Comput. Biol. Med..

[33] Radecka A, Pluta W, Lubkowska A (2021). Assessment of the dynamics of temperature changes in the knee joint area in response to selected cooling agents in thermographic tests. Int. J. Environ. Res. Public Health.

[34] Lahiri BB, Bagavathiappan S, Philip J (2020). Infrared thermal imaging based study of localized cold stress induced thermoregulation in lower limbs: The role of age on the inversion time. J. Therm. Biol..

[35] Ignacio Priego-Quesada J (2021). Reproducibility of skin temperature response after cold stress test using the game ready system: Preliminary study. Int. J. Environ. Res. Public Health.

[36] Benito-Martinez E (2020). Local and contralateral effects after the application of neuromuscular electrostimulation in lower limbs. Int. J. Environ. Res. Public Health.

[37] Litscher G, Wang L, Huang T, Zhang W (2011). Violet laser acupunctured part 3: Pilot study of potential effects on temperature distribution. J. Acupunct. Meridian Stud..

[38] Xu P, Cui S, Wee DAC, Sheng XU, Leang LT (2014). Preliminary observation on effect of cupping on the skin surface temperature of patients with back pain. World J. Acupunct. Moxib..

[39] Cage SA, Warner BJ, Gallegos DM (2020). Effect of cupping therapy on skin surface temperature in healthy individuals. J. Sports Med. Allied Health Sci..

[40] Formenti D (2013). Thermal imaging of exercise-associated skin temperature changes in trained and untrained female subjects. Ann. Biomed. Eng..

[41] Jones BF (1998). A reappraisal of the use of infrared thermal image analysis in medicine. IEEE Trans. Med. Imaging.

[42] Bernard V, Staffa E, Mornstein V, Bourek A (2013). Infrared camera assessment of skin surface temperature—Effect of emissivity. Phys. Med. Eur. J. Med. Phys..

[43] Fernandez-Cuevas I (2015). Classification of factors influencing the use of infrared thermography in humans: A review. Infrared Phys. Technol..

[44] Hou X (2021). Using laser Doppler flowmetry with wavelet analysis to study skin blood flow regulations after cupping therapy. Skin Res. Technol..

[45] Li T, Li Y, Lin Y, Li K (2017). Significant and sustaining elevation of blood oxygen induced by Chinese cupping therapy as assessed by near-infrared spectroscopy. Biomed. Opt. Express.

[46] Zaproudina N, Varmavuo V, Airaksinen O, Narhi M (2008). Reproducibility of infrared thermography measurements in healthy individuals. Physiol. Meas..

[47] Liu X (2020). Intra- and interrater reliability of infrared image analysis of facial acupoints in individuals with facial paralysis. Evid. Based Complement Altern. Med..

[48] Ludwig N, Formenti D, Gargano M, Alberti G (2014). Skin temperature evaluation by infrared thermography: Comparison of image analysis methods. Infrared Phys. Technol..

[49] Ludwig N (2016). Thermography for skin temperature evaluation during dynamic exercise: A case study on an incremental maximal test in elite male cyclists. Appl. Opt..

[50] Formenti D (2016). Dynamics of thermographic skin temperature response during squat exercise at two different speeds. J. Therm. Biol..

[51] Lahiri BB, Bagavathiappan S, Jayakumar T, Philip J (2012). Medical applications of infrared thermography: A review. Infrared Phys. Technol..

